# Boys Do Cry: Age and Gender Differences in Psycho-Physiological Distress during the COVID-19 Pandemic in Italy

**DOI:** 10.1007/s11482-022-10120-5

**Published:** 2022-11-15

**Authors:** Giulia M. Dotti Sani, Francesco Molteni, Simone Sarti

**Affiliations:** Department of Social and Political Sciences, University of Milan, Milan, Italy

**Keywords:** Psycho-physiological distress, COVID-19 pandemic, Death, Gender, Age, Italy

## Abstract

This article contributes to the quality of life literature by investigating gender and age gaps in psycho-physiological distress during the COVID-19 pandemic in Italy. Specifically, we investigate whether women experienced higher levels of distress than men, and if such gap can be explained by a greater negative reaction of women in the experience of a negative COVID-19 related event, such as the illness or death of a relative. Moreover, we explore whether age moderated or amplified the effect of a negative event on distress among women and men. To do so we rely on an ad hoc survey carried out between April 2020 and June 2021 in Italy, the first European country to be hit by the pandemic. Our results indicate that subjects who experienced the hospitalization or, more so, the death of a family member during the pandemic were more exposed to psycho-psychological distress compared to subjects who were not directly touched by COVID-19. Moreover, our results show that while women were on average more likely to express feelings of distress than men in absence of evident stressors, this gap disappeared among subjects who experience the death of a relative. Furthermore, our results indicate that experiencing a negative COVID-19 related event led to an increase in distress among all respondents except older men, who appeared to be the most resilient to the manifestation of any sign of distress. These results speak to important age and gender differences in the feelings and externalization of grief in the Italian context.

## Introduction

The recent COVID-19 pandemic has had a major impact on the lives of millions around the planet. Beyond the immediate health threat posed by the new coronavirus, women and men were also faced with other plague-related challenges, such as living though lockdowns, enduring long periods of isolation and social distancing, and coping with an indefinite state of economic uncertainty caused by the world being in the grip of the virus. Against this background, scholars and policy makers began expressing concern of what consequences the COVID-19 pandemic would have not only on the physical health of citizens around the globe, but also on their mental health. Seeking an answer to this question, a multitude of studies were carried out to investigate the effects of the pandemic on individual well-being.

Three empirical finding emerge consistently from this growing body of literature. First, studies show that proximity to the illness and the loss of a loved one to COVID-19 were associated with worsening mental health conditions and increasing levels of anxiety and depressive symptoms (Carson et al., 2021; Eisma & Tamminga, 2020; Pierce et al., 2020). While it was known prior to the pandemic that bereavement was linked with worsening mental health, recent studies suggest that these effects were amplified during COVID-19. Second, research shows that during the pandemic women were considerably more likely to encounter mental health issues (Arpino & Pasqualini, 2021; Bambra et al., 2021). Third, several studies indicated that young people suffered greater consequences in terms of well-being and mental health compared to older subjects (Lucchini et al., 2021; Maffly-Kipp et al., 2021). Overall, existing studies carried out during the pandemic suggest that experiencing a negative COVID-19 related event, being a woman, and being young are linked to worse mental health.

However, research has yet to address what happens at the *intersection* of gender, age, and the experience of a traumatic event. No one is just a woman, or a young person, or someone who went through the loss of a loved one. To the contrary, everyone is an expression of a *combination* of these (and other) characteristics that could potentially play different (and multiplicative) roles for certain outcomes, such as distress and well-being. It is plausible that living through a traumatic event might elicit different reactions (both in quality and quantity) among women compared to men. Similarly, youth might have a different reaction, for example, to the death of relative compared to an older individual. Ultimately, there might even be differences in the way the death of a relative affects the mental health and well-being of a young woman vs. a young man. In other words, different *combinations* of gender, age, and exposure to traumatic events might have additional and multiplicative effects on well-being that go unnoticed if we limit our focus to the *separate* effects of gender, age, and trauma. By ignoring the overlap between different individual characteristics, we risk to systematically underestimate or overestimate the actual effects of certain experiences on mental health. From a policy perspective, this is a critical issue, as identifying the most vulnerable sub-groups is crucial for constructing fine-tuned and targeted measures to address mental health issues (Arcaya et al., 2015; Fehrenbacher & Patel, 2020; Hankivsky et al., 2014).

This article contributes to the literature by investigating whether the experience of a traumatic COVID-19 related event, such as the death or hospitalization of a family member due to the illness, had a negative impact on well-being − measured as subjective psycho-physiological distress − and whether such impact varied at the intersection of age and gender of the respondents. Specifically, this article seeks to answer three key questions: 1) Are women who went through a traumatic event more likely than men to manifest issues of mental health? 2) When faced with a negative COVID-19 related event, do young respondents report more distress compared to older ones? 3) Lastly, is there an additional penalty paid by young women, who are at the intersection of two potentially vulnerable groups?

To address these questions, we rely on the ResPOnsE COVID-19 study, a Computer Assisted Web Interview (CAWI) survey carried out over two years of the pandemic in Italy. Italy was the first western country to be hit by the pandemic (Remuzzi & Remuzzi, 2020) and the confinement measures that were enacted to contain the spread of the virus were especially harsh on the population. These include an extensive period of total lockdown, the ongoing requirement to maintain social distancing and use of protective devices, and the prolonged closure of schools and businesses. Therefore, the country represents a valuable case study to evaluate the effects of a new and unknown threat to well-being.

As we will see later in this article, our results confirm previous findings by showing that facing a negative COVID-19 related event, being a woman, and being young were associated with worse outcomes in terms of mental health. However, we show that while women in general are worse off in terms of distress compared to men, the observed gender gap disappears among women and men who faced a negative COVID-19 related event, such as the death of a relative. In contrast, the difference between age groups becomes wider in the presence of a traumatic event, with young people experiencing considerably more distress compared to older subjects. Finally, our analyses reveal that when exposed to negative events, young men and women are equally vulnerable to distress, whereas older men are especially resilient to this expression of grief.

## Background

### Negative Events and Distress

An extensive scientific literature shows that accidental adverse events during the life course may be crucial factors of social stress (Aneshensel, 1992). Dismissal and unemployment, widowhood, being a victim of aggression, a serious illness of a family member or experiencing a catastrophic event (such as an earthquake) are some of the negative events that can undermine health and increase psychological stress. Unemployment, for example, is a stress factor that can debilitate the immune system, increase the probability of falling ill, and in general amplify the risk of worsening psychophysical well-being (Cohen et al., 2007a, 2007b; Sarti & Zella, 2016).

Among various sources of stress, scholars have attributed a critical role to the illness or loss of a loved one, such as a spouse, child, relative or friend. These events are associated with dramatic declines in life satisfaction and quality of life and can have negative and enduring consequences in terms of mental health and social functioning (Bonanno et al., 2004; Lamb et al., 2003; Lucas, 2007). Results by Ong et al. (2010) show that compared with continuously married controls, widowed women experienced a significant worsening of positive emotion in the years following the loss (even if there are differences among widows with respect to the relational conditions pre-existing the loss). A study by Liu and colleagues (Liu et al., 2019) shows negative and enduring consequences (such as a fall in vitality, worsening life satisfaction and lower perceived health) among individuals following the death of a close friend. Davidson et al. (2012) note the post-intensive care syndrome, namely, the development of negative psychological effects such as anxiety, acute stress disorder, posttraumatic stress, and depression that develop when a family member has a serious illness with an uncertain outcome.

The negative and enduring consequences of losing a loved one in terms of mental health and social functioning that were known already before the spread of the coronavirus are likely to have grown of salience in the recent COVID-19 pandemic. Many studies have showed increases in psycho-physiological distress during the pandemic, both in Italy and elsewhere (Forte et al., 2020; Mazza et al., 2020; Orgilés et al., 2020; Pierce et al., 2020). Recent research has found evidence of post-traumatic stress in subjects who lost a loved one during the pandemic and has also shown that a loss during the pandemic elicited more acute grief reactions compared to prior to the health emergency (Carson et al., 2021; Eisma & Tamminga, 2020; Mazza et al., 2020; McGinty et al., 2020). In a CAWI survey, Mazza and colleagues (Mazza et al., 2020) found that, in the earlier and harsher stages of the pandemic, when a family member was infected the interviewee on average presented higher levels of anxiety and stress. In another CAWI study on the consequences of death in the relational surrounding, Eisma and Tamminga (2020) showed that the circumstances of the bereavement during pandemic, such as relational isolation and in many cases the real absence of death rituals, aggravated both grief and psychological stress of the bereavement. Even the proximity of the illness was found to be negatively related to stress: a study by Su and colleagues (Su et al., 2020) conducted in Guangzhou, China, by means of an online survey of 403 residents found that the presence of individuals with COVID-19 in the same building was associated with higher anxiety levels.

Overall, researching the role of negative events linked to COVID-19 is crucial, because the illness not only represents a *direct* etiologic threat, but also an *indirect* risk factor capable of increasing psychological distress and compromising extensively the quality of life in a relational environment.

### Gender and Age Gaps in Feelings, Emotions, and Psycho-Physiological Distress

According to emotion scholars, societies are characterized by different “emotion cultures”. The emotion culture predominant in the US and other western countries holds that “women are both more emotional and more emotionally expressive than men” (Simon & Nath, 2004, p. 1137). Empirical studies in sociology and psychology do suggest that women report more negative feelings than men (Byles et al., 2012; Matud et al., 2015, 2022; Mirowsky & Ross, 1995) and recent studies show that women reported higher levels of distress also during the COVID-19 pandemic (Bambra et al., 2021). However, gender expectations may vary cross-culturally (Brebner, 2003; Olson et al., 2019). For example, a comparative study between German and Chinese samples shows that gender differences in self-perceived emotional intensity are more consistent with social norms and stereotypes in the Chinese sample, compared with the German one. The study indicates that such differences are neither constant nor universal, varying according to culture and age (Gong et al., 2018).

According to Parsons (1955, 1964), gender differences in feelings and in the expression of emotions are functional to the gendered division of labor in society: women need emotionality to fulfill their expressive roles, while men require unemotionality to best perform as breadwinners. Hochschild’s normative theory about emotion (1975, 1981) highlights how *feeling and expression norms* of the dominant emotion culture discourage men from feeling and expressing feelings in general, and certain feelings, such as sadness, in particular (just as much as they discourage women from feeling and expressing anger).

Developmental psychologists also stress that the gender gap in feelings may be more pronounced in the *expression of emotion* rather than in the *experience of emotion* (Brody & Hall, 1993; Kring & Gordon, 1998). In other words, at least part of the differences observed between the emotions of women and men would not be related to their actual feelings, but rather on the extent to which they externalized them. In fact, it has been argued that men are socialized to hide their feelings (i.e., boys don’t cry), whereas women “learn to express their emotions more freely.” (Simon & Nath, 2004, p. 1142). Moreover, predominant masculinity ideologies such as hegemonic masculinity (Connell & Messerschmidt, 2005) state that men should be stoic, controlled, and self-sufficient (Mahalik et al., 2003) and, indeed, from an early age, boys are taught to refrain from displaying signs of weakness and vulnerability (Vogel et al., 2011). In contrast to normative theories, structural theories argue that gender differences in feelings and their expression are determined by the status and power hold by individuals, regardless of their gender (Kemper, 1981; Ridgeway & Johnson, 1990; Risman, 1987).

Differences in feelings and the expression of emotions might also account for *age* gaps in the expression of emotions, well-being, and distress. The literature on ageing and well-being presents mixed results. Some studies suggest that positive affect improves with age (Carstensen et al., 2011; Gross et al., 1997), while others indicate that it remains stable (Hamarat et al., 2002; Kunzmann et al., 2013). Scholars have argued that part of these differences might occur because older respondents use different emotion regulation strategies to mitigate negative affect: “it is possible that older individuals do not necessarily report lesser negative emotions in the moment; they may simply retrospectively recall (relative to younger persons) experiencing fewer negative emotions.” (Hudson et al., 2016, p. 4). Moreover, it has been suggested that older male respondents might adhere more strictly to norms on hegemonic masculinity (Campos-Castillo et al., 2020; King et al., 2020) and hence be less likely to show negative affect, whereas younger male respondents might be less worried about displaying their feelings out of fear of being considered vulnerable (Vogel et al., 2011). Thus, differences between age groups could be imputed not only to processes that occur over the life course (i.e., age affects), but also to different ways in which people were socialized as children (i.e., cohort and generational effects).

Somewhat inconsistent findings emerge concerning the relationship among gender, age, and distress (Blanchflower & Oswald, 2008; Graham & Pozuelo, 2017). For example, in an Australian study, Phongsavan and colleagues (Phongsavan et al., 2013) found that older women experience a steeper reduction in the risk of psychological distress than men at age 65 to 74 and 75 to 84 compared to those aged 45 to 64. Byles et al. (2012) showed that the prevalence of psychological distress is lower at older ages but that women have higher distress than men even at older ages. The heterogeneity in results about the interaction between age and gender suggest a prominent role of the socio-cultural context molding different expectations, opportunities, and resources among age groups and between men and women (Cook, 1990; Mirowsky & Ross, 1995).

### Psycho-Physiological Distress during the COVID-19 Pandemic in Italy

Italy was the first western country to be hit by the pandemic (Remuzzi & Remuzzi, 2020) and is therefore the perfect context to evaluate the effects of a new and unknown threat on well-being. Indeed, multiple studies have aimed at monitoring the psychological well-being of Italians since the first weeks of the pandemic.

By means of an online survey administered to 2,766 individuals, Mazza et al. (2020) showed that women, negative affect, detachment, having a family member or an acquaintance infected, a history of stressful situations and medical problems, and being a young person who had to work outside their domicile were all factors associated with higher levels of depression, anxiety, and stress (to various degrees). By adopting a similar design, Forte et al. (2020) showed that respectively 31.38%, 37.19% and 27.72% of respondents reported levels of general psychopathological symptomatology, anxiety, and PTSD symptoms above the cut-off scores. In addition, they showed that being a woman, being under the age of 50, and having a direct contact with infected people were risk factors for psychological distress. Fiorillo et al. (2020) using data from the COvid Mental hEalth Trial (COMET, 20,720 participants) showed that 12.4% of respondents reported severe or extremely severe levels of depressive symptoms, 17.6% reported anxiety symptoms and 41.6% reported feeling at least moderately stressed by the situation. In addition, the authors found that women and people with pre-existing mental health problems were at higher risk of developing severe depression and anxiety symptoms. Rossi et al. (2020), using an online survey during the first lockdown in the spring of 2020, evaluated different outcomes such as post-traumatic stress symptoms (PTSS), depression, anxiety, insomnia, perceived stress, and adjustment disorder symptoms (ADS). The authors found that being a woman, being young and experiencing COVID-19 related stressful events were associated with all these outcomes. Quarantine, discontinued working activity, higher workload and having a loved one deceased of COVID-19 were also associated with many of these symptoms. Arpino and Pasqualini (2021), relying on a quota sample with post-stratification weights to obtain a representative sample of the Italian population with respect to key sociodemographic factors, found that 47% of respondents self-reported an increase in depressive feelings during the COVID-19 lockdown. Moreover, age, gender, and difficulties experienced during the first national lockdown were identified as the main determinants of such symptoms. The study by Lucchini and colleagues (Lucchini et al., 2021) based on the Italian panel ITA.LI is, to our knowledge, the only Italian study that relied on a pre-pandemic measure of psychological well-being. The authors found that mental health (measured on the 12-item Short-Form Health Survey) significantly worsened after the pandemic. Moreover, their results indicate that the fall in mental health was larger among women than among men, and among younger vs. older individuals.

### Contribution and Hypotheses

The research carried out during the first months of the COVID-19 pandemic in Italy indicates that age, gender, and proximity to the illness all played a significant role in psychological well-being. While these findings account for the effects of gender, age, and stressful event *separately,* these three characteristics come to co-exist within each individual. Therefore, building on the intersectionality approach in health research (Arcaya et al., 2015; Fehrenbacher & Patel, 2020; Hankivsky et al., 2014), we argue that investigating the *interaction effects* of age, gender, and exposure to COVID-19 negative events for psycho-physiological distress can significantly improve our understanding of mental health inequalities.

Based on previous research highlighting possible gender and age gaps in the expression of negative affect (King et al., 2020; Simon & Nath, 2004; Vogel et al., 2011), we test two sets of hypotheses. The first serves to confirm prior research on the association between gender, age, and negative events for distress (H1):

- H1a: Women report higher levels of distress during the pandemic than men
- H1b: Younger people report higher levels of distress during the pandemic than older ones
- H1c: Experiencing the death or hospitalization of a family member due to COVID-19 is associated with higher levels of psycho-physiological distress.

The second set of hypotheses sets out to test the presence of *interactions* between our predictors of interest. In particular, based on the notion that men in general and older men in particular will adhere to hegemonic masculinity standards and limit the expression of negative feelings in the face of adversity, we test the following hypotheses:

- H2a: Faced with a negative event, men report less distress than women.
- H2b: Faced with a negative event, older respondents report less distress than younger ones.
- H2c: Faced with a negative event, older men report the lowest levels of distress of all.

## Methodology

### Data and Sample

The analyses are based on three rounds of data from the ResPOnsE COVID-19 study (Italian Public Opinion Response to the Covid-19 Emergency), which aimed at monitoring changes in public opinion and well-being during the COVID-19 emergency in Italy.

The study collected daily information through online interviews (CAWI) in four surveys that took place between April 2020 and December 2021, for a total of over 30,000 interviews. The four waves of ResPOnsE COVID-19 were collected between April and July 2020 (Wave 1), in December 2020 (Wave 2), between March and June 2021 (Wave 3), and between November and December 2021 (Wave 4). The first and third wave follow a Rolling Cross-Section (RCS) design, in line with the dynamic nature of the pandemic phenomenon. The ResPOnsE COVID-19 study also includes a panel component, by which about 60% of subjects were interviewed twice (between the first and the third wave or between the first and the fourth wave). For further details on the research design,^1^ see the articles by Vezzoni et al. (2020) and Biolcati et al. (2021).

The reference population is made up of people aged 18 or over residing in Italy. Given the constraints of time and resources, it was not possible to resort to probabilistic procedures for the construction of the sample, opting instead for the selection of the names from an online community of a commercial research institute (SWG SpA). To correct for the expected distortions, the sample is stratified by macro-area of residence and made up of quotas based on gender and age. In addition, post-stratification weights are used to align the sample to the population.

From the overall sample we deleted three groups of subjects. First, we dropped the interviews carried out in Wave 2, when items on pyscho-physiological distress were not fielded altogether. Then, to avoid the distortions that would derive from including respondents that participated twice in the survey (i.e., the panel component), we randomly selected one observation for each respondent. Third, we applied listwise deletion to missing values on the variables used in the models, which correspond to 4.5% of the sample. This leaves us with a total of 17,060 respondents distributed in three different moments of the pandemic.

### Dependent Variable

Our dependent variable is a composite measure of psycho-physiological distress based on items suggested by the “COVID-19 and mental health measurement working group” at the Department of Mental Health Johns Hopkins Bloomberg School of Public Health (JHSPH). Specifically, we rely on four core mental health questions, which gauge how often in the past 7 days respondents have: 1) felt nervous, anxious, or on edge, 2) felt depressed, 3) felt lonely, and 4) had physical reactions, such as sweating, trouble breathing, nausea, or a pounding heart, when thinking about the experience with the coronavirus/COVID-19 pandemic (e.g., social distancing, loss of income/work, concerns about infection). Response options were: rarely or none of the time (less than 1 day); some or a little of the time (1–2 days); occasionally or a moderate amount of time (3–4 days); most or all the time (5–7 days). We used the items to generate an overall scale of psycho-physiological distress that, after rescaling, ranges from 0 to 10, where 0 indicates that the respondent never or rarely experienced the above feelings and 10 indicated a very high frequency of symptoms. The scale displays both high internal consistency (Cronbach alpha = 0.81) and one-dimensionality. In terms of validity, the literature discussed above highlights three main correlates of psycho-physiological distress during the pandemic: gender, age, and exposure to negative events. Consistent with these findings, psycho-physiological distress in our sample is found to be higher among women (one-way t =  − 23.04, Pr(T < t) = 0.000), younger respondents (18–34 years old, F = 348.17, Pr = 0.000), and those with a family member who died because of COVID-19 (F = 396.96, Pr = 0.000).

### Independent Variables

The main independent variables used in the study are gender (women vs. men) and age, which we construct as a categorical variable with three response options: 18–39, 40–54, and ≥ 55. Age groups were defined following two criteria. First, we aimed at constructing groups that were roughly equal in sample size. Second, because age differences in distress could also be imputed to *generational* differences, our age groups include respondents from three main birth cohorts: The older group (≥ 55) includes individuals born between 1926 and 1955. Of these, 90% were born after 1945 and thus belong the first and second cohort of Baby Boomers. The second age group (40 to 54) includes respondents belonging to Generation X, i.e., those born between 1966 and 1980, while the younger group (18–39) includes mostly Millennials (80%) and a smaller group of Generation Z (20%) (ISTAT 2016).^2^

Our third key independent variable is a measure that captures whether the respondent experienced a negative COVID-19 related event in the family network. The variable takes four possible outcomes: no event (reference category); a family member was infected by the virus; a family member was hospitalized due to COVID-19; a family member died with COVID-19. In case respondents experienced more than one negative event (e.g., a family member was hospitalized and then died), the most negative event is considered.

### Controls

Studies have shown that other factors are also relevant for psycho-physiological distress and well-being. However, following the approach of Bartram (2021), we refrain from including a standard set of controls in the models and aim at controlling only for potential *confounders*, i.e., variables that might be causally linked with both the dependent variable (distress) and the main independent variables of interest.

From this perspective, one variable that we identify as a potential confounder is time of the interview, which we include using a dummy per each of the 35 weeks of fieldwork. This control is relevant because time can affect not only the outcome of interest (distress) but also the likelihood of having experienced a negative COVID-19 related event. Since this results in many coefficients in the models (34 dummies), these are omitted from the presentation of the regression models in the main text but are reported in the Appendix. Similarly, it is plausible that geographical area of residence might also be a confounder and therefore we include it as a categorical variable (north-west as reference, north-east, center, south, islands). Especially in the first months of the pandemic, COVID-19 cases were more diffused in the north of the country. Therefore, respondents in this area might have been more likely to experience higher levels of distress and to have faced a negative COVID-19 related event.

Studies have also found that socio-economic groups differ in the extent to which they manifest distress: in particular, unemployment or having a low income are found to be associated with higher levels of stress (Sarti et al., 2021; Krieger et al., 2020; Li et al., 2021; Vinkers et al., 2020). Similarly, some studies have pointed toward an association between level of education and well-being (Ross & Wu, 1995). However, it is questionable to include these variables as controls because they might act as intervening variables, and not as confounders. For example, younger respondents might be more likely to be unemployed, and this might lead to a higher level of distress. To verify whether these variables intervene in the relationship between the main predictors of interest and the outcome, in preliminary analyses we included them in the models stepwise to verify their effect. Since we observed no difference in the outcomes of interest, we have deemed them “safe controls” and have included them in the models presented below. Specifically, we included level of education (lower secondary or less as reference, upper secondary, tertiary and above); employment status (employed as reference, retired, homemaker, unemployed, other) and a dummy variable gauging whether the household finds it difficult or very difficult to cope on current income. Summary statistics of the variables used in the models are presented in Table 1.

**Table 1:** Summary statistics – Means *(SD)* and proportions. *N* = *17,060*

	Men	Women
Psycho-physiological distress (min = 0, max = 10)	2.60 (*SD* = 2.42)	3.26 (*SD* = 2.62)
Age		
18–39	0.32	0.31
40–54	0.29	0.29
≥55	0.39	0.40
Family member experienced…		
No event	0.67	0.70
Infection	0.17	0.17
Hospitalization	0.05	0.04
Death	0.11	0.09
Level of education		
≤ Lower secondary	0.31	0.39
Upper secondary	0.46	0.41
≥ Tertiary	0.24	0.20
Employment status		
Employed	0.62	0.45
Retired	0.22	0.17
Homemaker	0.01	0.20
Unemployed	0.08	0.12
Other	0.08	0.07
Somewhat or very difficult on present income	0.49	0.58
Macro-area of residence		
North-East	0.27	0.27
North-West	0.20	0.19
Center	0.20	0.20
South	0.23	0.23
Islands	0.10	0.12
*N*	8830	8230

Source: Own calculation on ResPOnsE COVID-19 data, waves 1, 3, 4. Post-stratification weights are applied

### Modelling Strategy

We test our hypotheses using standard linear regression models. The first model tests our baseline hypotheses by including only the predictors of interest (namely gender, age, and experience of a negative event) while Model 2 also includes the controls. We then move to test our second set of hypotheses by means of interaction terms. In Model 3 we add the interaction between gender and the experience of a negative event, and in Model 4 we include the interaction between age and the experience of a negative event. Finally, Model 5 includes a three-way interaction between gender, age, and the negative event. Because interaction terms are complex to interpret, we graphically report Adjusted Predictions at the Means (APMs) with 95% confidence intervals. Moreover, in the text we also report Marginal Effects at the Means (MEMs) to test and discuss the existence of differences among the various groups of interest.

## Results

The results for the linear regression models testing our first set of (confirmatory) hypotheses are presented in Table 2 below. Model 1 includes only the predictors of interest. As can be seen, the coefficient for women is positive, indicating that they experienced greater levels of distress compared to men throughout the period (β = 0.69, *p* ≤ 0.000). Adjusting for other variables, the predicted value of distress (APMs) for men is 2.51 and 3.20 for women. Thus, we find support for H1a. We also find confirmation of H1b, as older subjects are found to have considerably lower levels of distress compared to their younger counterparts: the APMs of distress in the youngest age group is 3.41, and declines to 2.80 in the 40–54 category, and to 2.39 among the ≥55 category. Reverse adjacent contrasts indicate that the difference between each group and the one immediately younger are all statistically significant at the 5% level or lower. Finally, the coefficients signaling that a family member experienced a negative COVID-19 related event also go in the expected direction: subjects who were exposed to the death of a relative face considerably higher distress (APMs = 4.21) compared to those who faced a hospitalization (APMs = 3.16) and, less so, infection (APMs = 2.82) or no event (APMs = 2.63). The difference between each event and the adjacent, less severe one, are statistically significant at the 5% level, thus providing support for H1c. The coefficients from the model with controls (Model 2) do not diverge in a substantial manner from the ones in the uncontrolled model. Thus, overall, our data confirm previous findings indicating that women, younger subjects, and those who experienced a negative COVID-19 related event suffered higher levels of distress compared to men, older respondents, and those whose family members were not touched by the illness.

**Table 2:** Multivariate linear regression coefficients with standard errors in parentheses

	Model 1	Model 2	Model 3	Model 4
Women (r.c. men)	0.69***	0.63***	0.70***	0.64***
	(0.04)	(0.04)	(0.04)	(0.04)
Age group (r.c. 18–39)	–0.61***	–0.55***	–0.55***	–0.40***
40–54	(0.05)	(0.05)	(0.05)	(0.06)
	–1.02***	–0.86***	–0.86***	–0.71***
≥55	(0.04)	(0.05)	(0.05)	(0.06)
Family member… (r.c. no event)				
Infected	0.19***	0.17***	0.17**	0.21***
	(0.05)	(0.05)	(0.07)	(0.08)
Hospitalized	0.53***	0.54***	0.66***	0.60***
	(0.09)	(0.09)	(0.12)	(0.15)
Deceased	1.58***	1.59***	1.89***	2.31***
	(0.06)	(0.06)	(0.08)	(0.10)
Family member… × Gender				
Infected × Women			0.01	
			(0.10)	
Hospitalized × Women			–0.26	
			(0.18)	
Deceased × Women			–0.68***	
			(0.12)	
Family member… × Age group				
Infected × 40–54				–0.20
				(0.12)
Infected × ≥55				0.07
				(0.11)
Hospitalized × 40–54				–0.12
				(0.22)
Hospitalized × ≥55				–0.05
				(0.21)
Deceased × 40–54				–0.91***
				(0.15)
Deceased × ≥55				–1.54***
				(0.15)
Constant	2.87***	2.49***	2.45***	2.37***
	(0.04)	(0.12)	(0.12)	(0.12)
*N*	17,060	17,060	17,060	17,060
Sig	0.00	0.00	0.00	0.00
R–squared	0.09	0.13	0.13	0.13

Source: Own calculation on ResPOnsE COVID-19 data, waves 1, 3, 4. Dependent variable: psycho-physiological distress scale (0–10)

Models 2, 3, and 4 control for: week of interview, area of residence, difficulty coping on income, employment status, and level of education. r.c. = Reference category

Sig. level: *p* ≤ 0.10*; *p* ≤ 0.05**; *p* ≤ 0.01***

**Fig. 1:**
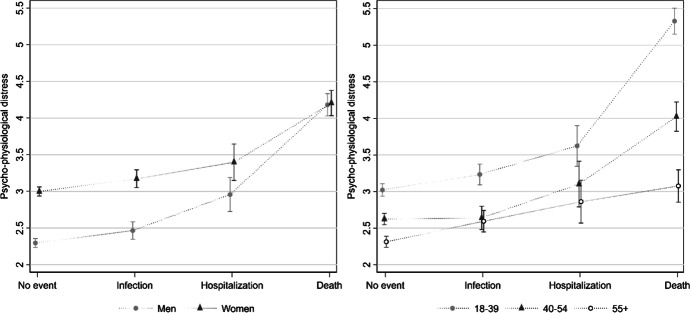
Predicted values (APMs) with 95% confidence intervals of pyscho-physiological distress by experience of COVID-19 negative event and gender (left) and age group (right). APMs are derived from Models 2 and 3 in Table 2

However, age, gender and their interaction might play a role in the way subjects react to adverse events. Thus, Table 2 also includes the results from the models with interactions between experiencing a negative event and, respectively, gender (Model 3) and age (Model 4). Starting from Model 3, the coefficients of the interaction terms go against H2a (i.e., Faced with a negative event, men experience less distress than women). Indeed, the negative coefficients indicate that the gender gap in distress is *smaller* among respondents experiencing the two most stressful events, i.e., the hospitalization and the death of a family member, and the difference is statistically significant in the latter case. Since the magnitude of the differences between groups (i.e., effect size) is complex to gauge when interaction terms are involved, we calculate and display in the left-hand side of Fig. 1 the Adjusted Predictions at the Means of psycho-physiological distress among women and men who experienced the different COVID-19 negative events. As can be seen, the APMs indicate that when they are *not* exposed to a (severe) threat women display significantly higher levels of distress than men (∆ = 0.70, p < 0.000). In contrast, when external conditions worsen, such as in the event of a having a family member who is hospitalized or dead, men experience a sharp increase in distress that brings them on par with women. In other words, under these circumstances the gender gap in distress closes. For example, men who experience the death of a family member have a predicted level of distress of 4.18 and women of 4.20 (∆ = 0.02, *p* > 0.10). This result is revealing, as it suggests that in the absence of major stressful event, women live with a baseline level of distress that is constantly higher than that of men.

Model 3 includes the interaction between experiencing a negative COVID-19 related event and age group to test hypothesis H2b (faced with a negative event, older people report less distress than younger ones). The negative and, in some cases, statistically significant coefficients for older age groups seem to suggest that the hypothesis is confirmed. However, it is more informative to resort to a visualization of the results. The right-hand side panel of Fig. 1 shows APMs with 95% confidence intervals for distress among the three age groups at each of the possible COVID-19 related events. The figure suggests that our hypothesis is confirmed: respondents in the youngest age group (18–39) not only report higher levels of distress than their older counterpart in the absence of negative events but are also the ones who experienced the steepest increase in distress when faced with negative external circumstances. Among this group (18–39), the predicted level of distress is 3.02 among those who experienced no negative event and 5.33 among those who experienced the death of a relative (*p* ≤ 0.000). The difference among the 40–54 year old group is somewhat less steep, going from 2.62 to 4.02 (*p* ≤ 0.000). In contrast, we find that older subjects react less to negative events, with their levels of distress ranging from 2.31 to 3.08 (*p* ≤ 0.000) among the ≥55 group. Overall, the results indicate that, as hypothesized, young subjects live with a higher baseline level of distress *and* respond worse to negative external circumstances.

**Fig. 2:**
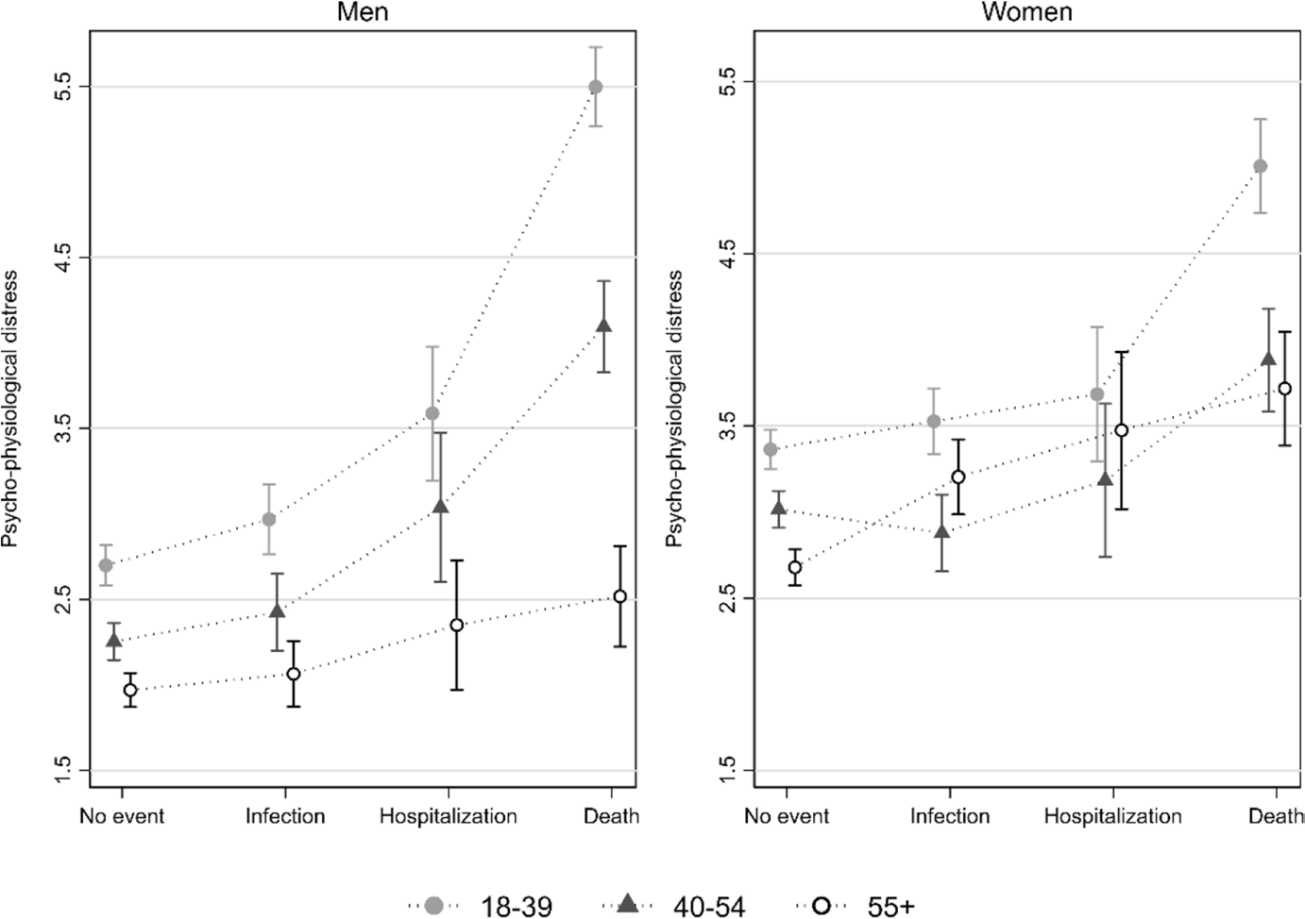
Predicted values (APMs) with 95% confidence intervals of pyscho-physiological distress by experience of COVID-19 negative event, gender, and age group. APMs are derived by the three-way interaction in Model 5 (see Appendix)

Finally, we ran Model 5 that included the three-way-interaction between age, gender, and the experience of a COVID-19 related event. The coefficients for the model are reported in the Appendix, while we rely on Adjusted Predictions at the Means and MEMs for the interpretation of the results. The predicted values are plotted in Fig. 2, separately by gender to simplify the presentation of the results. As we may notice from the graph, younger women and their male counterparts are both likely to experiences higher levels of distress in response to the death or the hospitalization of a relative due to COVID-19, with young women experiencing less distress than young men (5.00 vs. 5.5, MEM = -0.50, *p* ≤ 0.000). In contrast, in the absence of stressful events, young women aged 18–39 have a *higher* level of distress then young men (3.36 vs. 2.70, MEM = 0.66, *p* ≤ 0.000). Overall, the remarkable finding is that women and men in this age group are the ones who experience the highest levels of distress of all. The gender gap favors men in the absence of stressful events, while it favors women in case of severe negative events.

A similar pattern emerges among the 40–54 age group (Gen X), where we find women to be more distressed in the absence of negative events, while the gender gap is reversed in the face of adverse situations. Moreover, in case of the death of a relative, distress increases much more among men than among women.

But what about older women and men? Interestingly, women in the ≥55 group do not differ much in their levels of distress compared to their younger counterparts. They appear to be somewhat less distressed overall and to fare better than younger women in the absence of negative events, but they also experience a significant increase in distress when faced with the death of a relative. For example, women in the ≥55 age group who experienced the death of a family member see an increase in distress of 1.03 (MEM) compared to those who had no negative event (*p* ≤ 0.000). Overall, in generational terms, women from the boomer and X generation appear to be more resilient to negative events compared to Millennial women.

The situation is different among older men, who appear to be incredibly resistant to the manifestation of grief. Indeed, older men are not only the ones who display the lowest levels of distress in the absence of negative events (1.97 for men 55 and above): they are also the ones who experience the least increase in distress when faced with negative COVID-19 related events. Indeed, the APMs for a man ≥ 55 who experienced the death of a family member is 2.52, with an increase of only 0.55 compared to those who experienced no event (*p* ≤ 0.000). Moreover, in the face of death, older men experience significantly less distress compared to women in the same situation (MEM =  − 1.20, *p* ≤ 0.000). The difference with women in the same age group, but also with younger men, is highly suggestive of how the experience of grief and the ways of externalizing and communicating it are largely mediated by gender and by where one is in the life course. In particular, the large difference that emerges between the two younger group of men (18–39 and 40–54) and the older group (≥55) suggests that a generational shift might be happening in the way Italian men experience and manifest distress.

## Discussion and Conclusions

This article used original data collected during two years of the COVID-19 pandemic in Italy, the first European country to be hit by the new coronavirus, to examine gaps in psycho-physiological distress at the intersection of gender, age, and experience of a negative COVID-19 related event. While previous research has identified the *separate* effects on distress of these three predictors during the pandemic (Arpino & Pasqualini, 2021; Bambra et al., 2021; Carson et al., 2021; Eisma & Tamminga, 2020; Lucchini et al., 2021; Maffly-Kipp et al., 2021; Pierce et al., 2020), this article goes beyond the state of the art by focusing on the interaction effects of gender, age, and experience of a negative event for distress. By doing so, the study contributes to the quality of life literature from at least two perspectives. First, conceptually, by looking at different combinations of age, gender, and experience of a negative event, our study goes beyond the “one size fits all” approach that focuses on differences between broad social groups (e.g., women vs. men, young vs. old), and acknowledges instead the distinctive role played by both within and between-group differences for individual outcomes. Although far from being fully accurate, this approach highlights that there is significant information to be gained if we focus on the interplay of the different components of individual identity and experience, instead of dissecting and analyzing them separately. Second, “unpacking” the different contributions of gender, age, and negative experience for pyscho-physiological distress allows us to achieve a clearer and fine-grained picture of which subjects are most likely to experience negative affect. Indeed, previous studies on health outcomes have highlighted how the intersectionality approach can significantly improve our understanding of health inequalities (Arcaya et al., 2015; Fehrenbacher & Patel, 2020; Hankivsky et al., 2014), consequently enabling policy makers to develop fine-tuned instruments to better address the mental health needs of different sub-populations.

Our results confirm previous findings by indicating that, during the COVID-19 pandemic, younger respondents, women, and those who experienced the hospitalization or death of a relative were more exposed to psycho-physiological distress compared to older respondents, men, and those whose relatives were not exposed to the illness. More importantly, the results indicate the existence of an *interplay* between the three variables. First, our analyses show that while women experienced higher distress in the absence of “trigger” events, the gender gap disappeared when a very negative event occurred: women and men who experienced the hospitalization or death of a relative displayed the same level of distress. Second, our data showed that younger respondents were overall more distressed than older ones, but on average the age gap *increased* in case of a negative event. In other words, we observed a chasm between older and younger respondents in reaction to grief (King et al., 2020; Simon & Nath, 2004; Vogel et al., 2011). The former result could be explained by the fact that many younger subjects might have experienced the death of a relative for the first time during the COVID-19 pandemic. Generally, because they have lived less, younger respondents have had less occasions to deal with grief and therefore might react worse compared to older subjects, who are likely to have already suffered the loss of significant others in the past. However, the gap may also be sign of a generational change in the extent to which Millennials are more likely to express their feelings and emotions compared their predecessors. Third, when we zoom in on the interaction between age, gender, and experience of a negative event, the results indicate that such chasm is mostly confined to men. In fact, among women, for each increment in the severity of the negative event we observe an increase in distress that is common, though not identical, among all age groups: younger women were slightly more exposed to distress, but the differences are, overall, limited. In contrast, among men who experienced the death of a relative, we find a large increase in distress in the younger age groups, and a much smaller increase among the eldest group.

These results could be interpreted as a result of possible generational changes in the way Italian men adhere to traditional masculine norms by which men are expected to be stoic, impervious to pain and unfazed by hardship (McVittie et al., 2017). Compared to male baby boomers, younger men belonging to the Millennial or X generation, might not only be actually more distressed, but also more incline to externalize their feelings of distress, even if this means showing their vulnerability (Pederson & Vogel, 2007). These results would be in line with research from other fields that show how Italian men are becoming progressively more active in family life and are embracing roles that were traditionally considered feminine such as caregiving (Dotti Sani & Treas, 2016; Zajczyk & Ruspini, 2008).

Some limitations of the research are worth pointing out. First, the research initiated at the very beginning of the COVID-19 pandemic, thus at a time of great uncertainty and rapid changes. Under these circumstances, it was not possible to recruit participants through strictly probabilistic sampling procedures. With the aim of getting in the field as early as possible to document the effects of the pandemic on the population, we resorted to an online commercial panel. Thus, the results are not strictly speaking generalizable to the wider Italian population. Nonetheless, the use of quotas, stratification and weights guarantees that the distribution of our sample resembles that of the population. Second, as we focus only on one country, our results are limited to this single context. Future research could fruitfully address whether the gender and age/generational gaps we observe are found also in other countries. Third, our research is carried out during a unique period of human history – two years of the COVID-19 pandemic – and it is possible that the stark between and within-group differences that we find might have been exacerbated by the dramatic circumstances under which they were captured. Fourth, our dependent variable captures one specific aspect of well-being and quality of life: psycho-physiological distress. Future studies are needed to evaluate gender and age gaps in other domains, such as happiness or life satisfaction, at times of great personal and/or societal stress. Finally, our research design impedes achieving definite answers on whether the differences among age groups that we find are, actually, age effects or rather cohort/generational effects. Answering this question is beyond the scope of this study, but since, theoretically, both mechanisms could be at play, more research is needed to understand whether the “resilience to pain” that we observe in older men occurs because they became tougher with age, or because they were socialized at a time when boys did not cry.

To conclude, our study has contributed to the quality of life literature by unveiling important differences in the experience of distress among women and men of different age groups and experiencing varying degrees of negative events. This approach, which focused on the manifestation of distress at the intersection of various individual characteristics, can be of aid to policy makers when developing measures to target mental health inequalities. In particular, acknowledging the high levels of distress experienced by women generally and by young men and women who lost a loved one during the pandemic can orient policy tools to aid these specific groups of subjects. Moreover, as men are known to be less likely to seek psychological help than women (Lynch et al., 2018; Yousaf et al., 2015), it is critically important that policy makers are aware of the malaise experienced by this particular group. Indeed, the mental health of groups that are less likely to voice their distress (older men) should be kept into account, as well of the one of groups that are found to chronically report higher levels of distress, such as women. Hence, we recommend the development of policies to continuously monitor the social and mental health situation of the general population in times of societal upheaval, such as a greater empowerment of general practitioners (GPs) to observe the physical and mental health of their patients, and through national and local level policies and campaigns reminding citizens to be proactive in looking after their mental health. The COVID-19 pandemic has taken an enormous toll on the mental health of citizens across the globe. It is time for governments to recognize this and provide national health care systems with greater economic resources to specifically target mental health care issues.

## Appendix

Table 3.

**Table 3:** Multivariate linear regression coefficients with standard errors in parentheses

	Model 1	Model 2	Model 3	Model 4	Model 5
Women (r.c. men)	0.69***	0.63***	0.70***	0.64***	0.66***
	(0.04)	(0.04)	(0.04)	(0.04)	(0.08)
Age group (r.c. 18–39)					
40–54	–0.61***	–0.55***	–0.55***	–0.40***	–0.45***
	(0.05)	(0.05)	(0.05)	(0.06)	(0.08)
≥55	–1.02***	–0.86***	–0.86***	–0.71***	–0.73***
	(0.04)	(0.05)	(0.05)	(0.06)	(0.08)
Family member… (r.c. no event)					
Infected	0.19***	0.17***	0.17**	0.21***	0.27**
	(0.05)	(0.05)	(0.07)	(0.08)	(0.12)
Hospitalized	0.53***	0.54***	0.66***	0.60***	0.89***
	(0.09)	(0.09)	(0.12)	(0.15)	(0.21)
Deceased	1.58***	1.59***	1.89***	2.31***	2.80***
	(0.06)	(0.06)	(0.08)	(0.10)	(0.13)
Gender × negative event					
Infected × Women			0.01		–0.10
			(0.10)		(0.16)
Hospitalized × Women			–0.26		–0.57*
			(0.18)		(0.29)
Deceased × Women			–0.68***		–1.15***
			(0.12)		(0.20)
Age group × negative event					
Infected × 40–54				–0.20	–0.10
				(0.12)	(0.17)
Infected × ≥55				0.07	–0.17
				(0.11)	(0.16)
Hospitalized × 40–54				–0.12	–0.10
				(0.22)	(0.31)
Hospitalized × ≥55				–0.05	–0.51*
				(0.21)	(0.29)
Deceased × 40–54				–0.91***	–0.96***
				(0.15)	(0.20)
Deceased × ≥55				–1.54***	–2.25***
				(0.15)	(0.20)
*Age × Gender*					
40–54 × Women					0.10
					(0.11)
≥55 × Women					0.05
					(0.11)
Negative event × Age group × Women					
Infected × 40–54 × Women					–0.20
					(0.24)
Infected × ≥55 × Women					0.53**
					(0.23)
Hospitalized × 40–54 × Women					–0.05
					(0.44)
Hospitalized × ≥55 × Women					0.98**
					(0.43)
Deceased × 40–54 × Women					0.18
					(0.29)
Deceased × ≥55 × Women					1.64***
					(0.31)
Macro–area of residence (r.c. North–East)					
North–West		0.03	0.02	0.02	0.01
		(0.05)	(0.05)	(0.05)	(0.05)
Center		0.06	0.07	0.06	0.07
		(0.05)	(0.05)	(0.05)	(0.05)
South		0.38***	0.38***	0.39***	0.39***
		(0.05)	(0.05)	(0.05)	(0.05)
Islands		0.27***	0.27***	0.27***	0.26***
		(0.07)	(0.07)	(0.06)	(0.06)
Difficult on present income		0.69***	0.69***	0.69***	0.69***
(r.c. not difficult)		(0.04)	(0.04)	(0.04)	(0.04)
Employment status (r.c. Employed)					
Retired		–0.06	–0.06	–0.08	–0.06
		(0.06)	(0.06)	(0.06)	(0.06)
Homemaker		–0.09	–0.09	–0.10	–0.13*
		(0.08)	(0.08)	(0.08)	(0.08)
Unemployed		0.53***	0.52***	0.52***	0.53***
		(0.07)	(0.07)	(0.07)	(0.07)
Other		0.61***	0.61***	0.64***	0.64***
		(0.08)	(0.08)	(0.08)	(0.08)
Level of education (r.c. ≤ Lower secondary)					
Upper secondary		–0.15**	–0.15**	–0.15**	–0.14**
		(0.06)	(0.06)	(0.06)	(0.06)
≥ Tertiary		–0.06	–0.06	–0.04	–0.03
		(0.07)	(0.07)	(0.07)	(0.07)
Week of the interview					
13 Apr – 19 Apr (2020)		–0.07	–0.06	–0.06	–0.04
		(0.12)	(0.12)	(0.12)	(0.12)
20 Apr – 26 Apr (2020)		0.03	0.04	0.04	0.05
		(0.12)	(0.12)	(0.12)	(0.12)
27 Apr – 03 May (2020)		0.11	0.11	0.11	0.12
		(0.12)	(0.12)	(0.12)	(0.12)
04 May – 10 May (2020)		0.06	0.07	0.06	0.08
		(0.13)	(0.13)	(0.12)	(0.12)
11 May – 17 May (2020)		0.01	0.02	0.02	0.04
		(0.13)	(0.13)	(0.13)	(0.13)
18 May – 24 May (2020)		–0.13	–0.13	–0.13	–0.12
		(0.12)	(0.12)	(0.12)	(0.12)
25 May – 31 May (2020)		–0.16	–0.15	–0.15	–0.14
		(0.12)	(0.12)	(0.12)	(0.12)
01 June – 07 June (2020)		–0.12	–0.11	–0.10	–0.09
		(0.12)	(0.12)	(0.12)	(0.12)
08 June– 14 June (2020)		–0.34***	–0.33***	–0.34***	–0.33***
		(0.12)	(0.12)	(0.12)	(0.12)
15 June– 21 June (2020)		–0.31**	–0.30**	–0.30**	–0.29**
		(0.12)	(0.12)	(0.12)	(0.12)
22 June– 28 June (2020)		–0.13	–0.13	–0.12	–0.10
		(0.12)	(0.12)	(0.12)	(0.12)
29 June– 05 July (2020)		–0.36***	–0.35***	–0.35***	–0.34***
		(0.12)	(0.12)	(0.12)	(0.12)
06 July – 09 July (2020)		–0.21*	–0.21*	–0.21*	–0.21*
		(0.12)	(0.12)	(0.12)	(0.12)
15 Mar – 21 Mar (2021)		0.47***	0.48***	0.47***	0.49***
		(0.18)	(0.18)	(0.18)	(0.18)
22 Mar – 28 Mar (2021)		–0.29*	–0.30*	–0.27*	–0.27*
		(0.16)	(0.16)	(0.16)	(0.16)
29 Mar – 04 Apr (2021)		–0.03	–0.02	–0.00	0.02
		(0.16)	(0.16)	(0.16)	(0.16)
05 Apr – 11 Apr (2021)		–0.11	–0.11	–0.11	–0.08
		(0.15)	(0.15)	(0.15)	(0.15)
12 Apr – 18 Apr (2021)		0.12	0.12	0.15	0.16
		(0.15)	(0.15)	(0.15)	(0.15)
19 Apr – 25 Apr (2021)		–0.14	–0.14	–0.13	–0.13
		(0.15)	(0.15)	(0.15)	(0.15)
26 Apr – 02 May (2021)		–0.02	–0.01	–0.01	0.00
		(0.15)	(0.15)	(0.15)	(0.15)
03 May – 09 May (2021)		0.19	0.19	0.21	0.20
		(0.16)	(0.16)	(0.16)	(0.16)
10 May – 16 May (2021)		0.14	0.15	0.16	0.18
		(0.16)	(0.16)	(0.16)	(0.15)
17 May – 23 May (2021)		–0.01	–0.01	0.01	0.02
		(0.15)	(0.15)	(0.15)	(0.15)
24 May – 30 May (2021)		–0.06	–0.06	–0.05	–0.04
		(0.15)	(0.15)	(0.15)	(0.14)
31 May – 06 June (2021)		–0.03	–0.04	–0.02	–0.03
		(0.15)	(0.15)	(0.15)	(0.15)
07 June– 13 June (2021)		0.01	0.03	0.01	0.03
		(0.15)	(0.15)	(0.15)	(0.15)
14 June– 20 June (2021)		0.01	0.02	–0.03	–0.02
		(0.21)	(0.21)	(0.21)	(0.21)
8 Nov – 14 Nov (2021)		–0.26	–0.24	–0.22	–0.18
		(0.19)	(0.19)	(0.19)	(0.19)
15 Nov – 21 Nov (2021)		–0.06	–0.06	–0.03	–0.04
		(0.16)	(0.16)	(0.16)	(0.16)
22 Nov – 28 Nov (2021)		–0.24	–0.23	–0.25	–0.25
		(0.16)	(0.16)	(0.16)	(0.16)
29 Nov – 05 Dic (2021)		–0.14	–0.14	–0.13	–0.12
		(0.16)	(0.16)	(0.16)	(0.15)
06 Dic – 12 Dic (2021)		–0.59***	–0.58***	–0.57***	–0.57***
		(0.16)	(0.16)	(0.16)	(0.16)
13 Dic – 19 Dic (2021)		–0.22	–0.22	–0.17	–0.16
		(0.19)	(0.19)	(0.19)	(0.19)
20 Dic – 26 Dic (2021)		–0.76	–0.77	–0.75	–0.72
		(0.83)	(0.83)	(0.82)	(0.82)
Constant	2.87***	2.49***	2.45***	2.37***	2.33***
	(0.04)	(0.12)	(0.12)	(0.12)	(0.13)
Sig	0.00	0.00	0.00	0.00	0.00
R–squared	0.09	0.13	0.13	0.13	0.14

Source: Own calculation on ResPOnsE COVID-19 data, waves 1, 3, 4. Dependent variable: psycho-physiological distress scale (0–10). *N* = 17,060

## Footnotes

1 Further information on the dataset is available in Italian here. The data in Italian can be downloaded here.

2 In preliminary analyses we tested alternative grouping strategies using four groups or by dropping the oldest and youngest respondents, i.e., the so-called Rebuild Generation (born before 1945, analogues of the silent generation in the US) and Gen Z. Results were robust to these various alternatives and are available upon request from the authors.
